# Brain Structural and Functional Substrates of Personal Distress in Empathy

**DOI:** 10.3389/fnbeh.2018.00099

**Published:** 2018-05-15

**Authors:** Siyang Luo, Shengqi Zhong, Yiyi Zhu, Cong Wang, Junkai Yang, Li Gu, Yingyu Huang, Xiaolin Xie, Shaofeng Zheng, Hui Zhou, Xiang Wu

**Affiliations:** ^1^Department of Psychology, Guangdong Key Laboratory of Social Cognitive Neuroscience and Mental Health, Sun Yat-sen University, Guangzhou, China; ^2^Guangdong Provincial Key Laboratory of Brain Function and Disease, Sun Yat-sen University, Guangzhou, China

**Keywords:** personal distress, empathy, voxel-based morphometry, functional connectivity, MRI

## Abstract

Empathy is the capacity to understand and experience the feeling state of others. While individuals attribute negative empathic responses to their own feelings, they would endure personal distress that can be harmful to social interaction. However, the neural mechanism of personal distress remains unclear. Here, we examined the neural substrates of personal distress by combining structural (Voxel-based morphometry (VBM)) and functional (resting-state functional connectivity (FC) analysis) MRI approaches in 53 college students (aged 19–26). A negative correlation was found between a trait measure of personal distress and gray matter (GM) volume in the dorsal medial prefrontal cortex (dmPFC). FC analyses with the dmPFC as a seed further revealed that the connectivity between the dmPFC and posterior insula was positively correlated with the personal distress, and the connectivities between the dmPFC and the anterior middle cingulate cortex, left lateral frontal cortex, and left inferior parietal gyrus were negatively correlated with the personal distress. Our results suggested that personal distress is underlain by neural substrates associated with both cognitive and affective mechanisms. Taken together, the structural and functional correlates of personal distress revealed in the present findings shed new light into the understanding of empathy.

## Introduction

Empathy refers to the ability to understand, infer and experience the feeling state of others in interpersonal interaction. As a kind of empathic response, personal distress is defined as a “self-oriented” feeling of personal unease to another’s state (Davis, 1980, 1983). Unlike sympathy or empathic concerns that is associated with prosocial urge to help others, personal distress is associated with negative affect and a series of problems in social interaction (e.g., abuse in parents and compassion fatigue in clinical workers; Batson et al., 1987; de Paúul et al., 2008; Thomas, 2013).

Empathy is suggested to be composed of two components, affective sharing and cognitive evaluation. Affective sharing is responsible for making emotional responses to another’s states, while cognitive evaluation is dedicated to inferring and understanding other’s mental state. Neuroimaging studies have found activities in the anterior insula (AI) and anterior cingulate cortex (ACC) when participants empathizing pain and emotion in others and these activities are related to participants’ subjective feelings, which reflects the affective component of empathy (Wicker et al., 2003; Singer et al., 2004, 2006, 2008; Jabbi et al., 2007, 2008; Lamm et al., 2007, 2011; Prehn-Kristensen et al., 2009). On the other hand, researchers found that the cognitive component of empathy involves at least two types of capacity, the ability to understand others’ mental states and the ability to distinguish between the observer and observed (Decety and Meyer, 2008; Preston and Hofelich, 2012), which rely on mechanisms underlying the Theory of Mind (ToM; Decety, 2011). A “Theory of Mind” or “mentalizing” network is suggested to be responsible for inferring mental states of others base on self-related and other-related social information (Mitchell, 2009). Abu-Akel (2003) stated that “Several neurobiological models have been proposed as based for ToM. These models and many others have invoked structures in posterior and anterior regions of the brain most consistently being the superior temporal sulcus and the medial prefrontal cortex, respectively. Limbic-paralimbic structures have also been suggested as part of the ToM circuitry most notably being the orbitofrontal cortex and the amygdala.” Using meta-analysis, researchers suggested that the dorsal medial prefrontal cortex (dmPFC) and bilateral temporo-parietal junction (TPJ) are consistently activated in ToM tasks, and regions including the precuneus, inferior frontal gyrus (IFG), precentral gyrus, ACC, temporal pole, posterior dmPFC and ventral mPFC are also reliably activated (Lamm et al., 2011; Molenberghs et al., 2016).

Studies using task-fMRI have reported that affective processing in the ACC and AI was associated with personal distress (Lawrence et al., 2006; Cheetham et al., 2009). However, personal distress might also be related with cognitive empathy. One important factor inducing personal distress is that people fail to maintain the boundaries between self and others, and as a result, individuals wrongly attribute the affective responses induced by empathy to others or to oneself (Lamm et al., 2016). Specially, ToM is thought to be important for realizing the difference between others and self (Gallagher and Frith, 2003). Activation of the dmPFC—a region essential for ToM, was repeated found more than 35 times in 40 ToM studies in a meta-analysis (Carrington and Bailey, 2009), and was found to be stronger in other-relevant than in self-relevant tasks (Murray et al., 2012). Thus, in the context of empathy, the lack of the ability distinguishing between self and others might be related to brain regions involved in cognitive empathy.

In addition, despite the findings of personal distress-related brain activities in previous task-based functional brain-imaging studies, relatively little is known about whether and how individual differences in trait measure of personal distress are associated with variation in brain structure and resting-state functional connectivity (FC). In particular, while task-based fMRI emphasizes investigation of brain activity when participants explicitly focus on specific tasks, in the past decades progresses in brain-imaging research have revealed the importance of using resting-state fMRI to investigate intrinsic brain activity when participants are not focused on a specific task (Gusnard and Raichle, 2001; Lowe, 2012). A better understanding of neural substrates of a cognitive function requires adopting both task-based fMRI with specific tasks related to the function and resting-state fMRI with non-task assessments of the function, such as the trait measure. We hypothesized that individual differences in personal distress could be reflected in gray matter (GM) volume variation in brain regions related to cognitive empathy, especially those indicated in ToM processing; and in variation in functional connectivities between cognitive- and affective-related regions. To this end, the current study assessed a trait measure of personal distress (Davis, 1994) in a group of participants from Sun Yat-sen University and acquired their structural and resting-state functional MRI data. Gray matter volume was examined using the Voxel-based morphometry (VBM) method (Ashburner and Friston, 2000), and FC was investigated using the seed-based analysis approach (Friston, 1994).

## Materials and Methods

### Participants

The present study recruited 54 participants from Sun Yat-sen University, China. One participants were excluded for missing behavioral data, and 53 participants (39 females, 14 males; age: *M* = 21.79, SD = 1.6) were included in the subsequent analyses. All participants indicated that they did not have history of neurological or psychiatric disorders, sensorimotor or cognitive impairment, or other anatomical injuries of brain, by completing pre-scanning self-reported questionnaires. Before conducting the study, informed consent was obtained from all the participants and possible consequences of the studies were explained, and this study was approved by the Institutional Review Board in the Department of Psychology of Sun Yat-sen University.

### Image Acquisition

All participants were scanned on a Siemens 3.0 Tesla MRI scanner (Siemens, Erlangen, Germany) at South China Normal University (Guangzhou, China). We used headphones and foam pads to avoid interference of scanner noise and reduce participants’ head motion in the scan. Participants were instructed to close their eyes, clear their thoughts but not to fall asleep, and move as little as possible during the data acquisition. Structural images of T1-weighted images covering the entire brain were obtained in a sagittal orientation by employing magnetization prepared by rapid gradient echo sequence (MPRAGE) : repetition time (TR) = 2300 ms, echo time (TE) = 3.24 ms, flip angle (FA) = 9°, field of view (FOV) = 256 × 256 mm^2^, inversion time = 900 ms, matrix = 256 × 256, slices = 176, slice thickness = 1 mm and voxel size = 1 × 1 × 1 mm^3^. Whole brain T2*-weighted resting-state functional images were acquired for 8 min using an echo-planar imaging (EPI) sequence: TR = 2000 ms, TE = 30 ms, FA = 90°, FOV = 224 × 224 mm^2^, slices = 32, matrix = 64 × 64, slice thickness = 3.5 mm, voxel size = 3.5 × 3.5 × 3.5 mm^3^, 240 volumes, and interleaved slice ordering.

### Behavioral Assessment

Participants completed a questionnaire that included the demographic information, and the trait measure of personal distress using the Interpersonal Reactivity Index (IRI; Davis, 1994). The personal distress measure included seven items. An example item was: “Being in a tense emotional situation scares me.” Participants answered to these items on a 5-point Likert scale (0 = strongly disagree, 4 = strongly agree). The higher total scores indicate stronger personal distress.

### VBM Analysis

The structural MRI data were processed using Statistical Parametric Mapping (SPM12^1^) and Computational Anatomy Toolbox (CAT12; r1073^2^). Processing consisted of standard VBM processing procedures as implemented in CAT12. T1 images were segmented and normalized and modulated GM images were obtained, according to the Diffeomorphic Anatomical Registration using Exponentiated Lie algebra (DARTEL) template from 555 healthy control subjects in the IXI-database. The voxel size was 1.5 × 1.5 × 1.5 mm^3^. Subsequently, the GM images were smoothed with an 8 mm full-width at half-maximum (FWHM) smoothing kernel.

Regression analyses were then conducted to examine the association between personal distress scores and GM volumes using SPM12. Significant GM volumes associated with the personal distress scores were identified using a voxel level threshold of *P* < 0.001 and a cluster level threshold of *P* < 0.05 (familywise-error corrected for multiple comparisons). A GM mask (voxels with a 30% or more likelihood of being situated in the GM were used to compute a binary GM mask) was applied as the inclusive mask during the analyses.

### Seed-Based Functional Connectivity Analysis

Rest-fMRI data preprocessing was then conducted by SPM8^3^ and Data Processing Assistant for Resting-State fMRI (DPARSF; Chao-Gan and Yu-Feng, 2010). Preprocessing consisted of standard resting-state FC preprocessing procedures as implemented in DPARSF, including removing the first 10 volumes of functional images, slice timing correction, motion correction (Luo et al., 2015), coregistration of structure images to functional images, segmentation with the DARTEL method (Ashburner, 2007), normalization to the standard MNI space with the DARTEL method and resampling functional images at a voxel size of 3 × 3 × 3 mm^3^, removing linear trends, regressing out nuisance variables (24 head motion parameters, white matter signals, and cerebrospinal fluid signals), filtering (0.01–0.08 Hz), and spatial smoothing (8-mm FHWM). The clusters identified in the VBM analysis were defined as regions-of-interest (ROIs) and served as the seeds in the seed-based FC analysis. For each participant, the time courses of voxels in each ROI were extracted and averaged across voxels. Linear (Pearson) correlation was computed between seed time series and time series of other voxels in the brain and the correlation coefficients were transformed into Fisher’s Z-scores. After that, regression analyses were conducted to examine the association between the seed-based functional connectivities and personal distress scores. Significant functional connectivities associated with the personal distress scores were identified using a voxel level threshold of *P* < 0.005 and a cluster level threshold of *P* < 0.05 (familywise-error corrected for multiple comparisons).

Note that structural images had higher spatial resolution than functional images and accordingly the voxel size was smaller in the VBM analysis (1.5 × 1.5 × 1.5 mm^3^) than in the FC analysis (3 × 3 × 3 mm^3^). In order to reduce the risk of false positive results partly due to smaller voxel sizes, Mueller et al. (2017) have suggested to adopt stricter statistical criteria for smaller voxel sizes. Therefore, at the voxel level, *P* < 0.005 was used in the FC analysis (Ko et al., 2015) and a more conservative criterion of *P* < 0.001 was used in the VBM analysis in the present study (Mueller et al., 2017).

## Results

The reliability of the trait measure of personal distress was 0.72. Females got marginally higher (*p* = 0.09) personal distress scores (14.41 ± 3.50) than males (12.62 ± 3.18), which was consistent with previously reported sex difference on personal distress (Davis, 1980).

**Figure 1:**
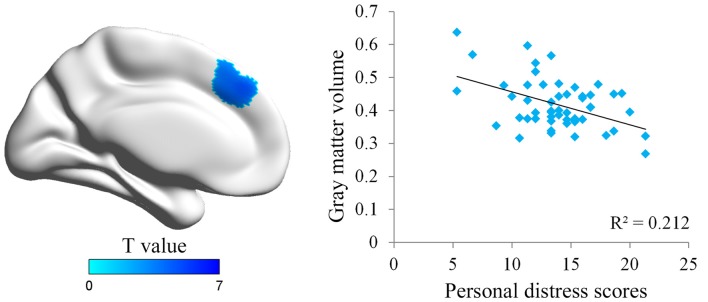
Illustration of the voxel-based morphometry (VBM) results. The personal distress was negatively associated with gray matter (GM) volume in dorsal medial prefrontal cortex (dmPFC). Scatter plot of the negative association between individuals’ personal distress scores and GM density values in the dmPFC cluster.

We first conducted whole brain regression analysis to investigate the association between participants’ personal distress scores and their GM volume. We included age, gender and total GM volume as covariates of no interest in design matrix to regress out any extraneous effects of them. The results showed that personal distress was negatively correlated with GM volume within a cluster in dmPFC (MNI coordinates: −8, 38, 39; cluster size: 617), indicating that individuals with less volume in dmPFC tended to experience personal distress during empathy (Figure 1 and Table 1). Similar results were observed without controlling age, gender and total GM volume. No significant positive correlation was observed in the VBM analysis.

**Table 1:** Association between personal distress and gray matter (GM) in dorsal medial prefrontal cortex (dmPFC).

					MNI coordinate		
	Regions	Correlation	*k*	*T*	*x*	*y*	*z*
Negative correlation	dmPFC	Negative	944	4.71	−8	38	39
	Superior occipital gyrus	Negative	227	3.84	−42	−75	29
Negative correlation^a^	dmPFC	Negative	617	4.10	−8	38	39

*^a^Results with gender and age as covariants*.

**Figure 2:**
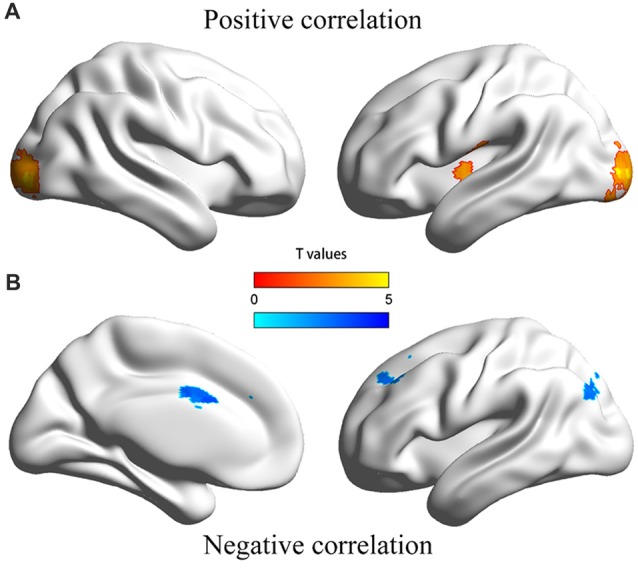
Illustration of the correlation between personal distress and functional connectivities between dmPFC and other regions. **(A)** Personal distress scores were positively correlated with functional connectivities of the dmPFC with the left posterior insula and occipital cortex. **(B)** Personal distress scores were negatively correlated with functional connectivities of the dmPFC with the anterior middle cingulate cortex, dorsal lateral prefrontal cortex and inferior parietal gyrus.

The dmPFC region found in VBM analysis was then used as the seed in the seed-based FC analysis of resting-state functional MRI data. Significantly positive correlations were found between personal distress scores and strength of FCs of the dmPFC with the left posterior insula and bilateral occipital gyrus, and significantly negative correlations were found between personal distress scores and strength of FCs of the dmPFC with the anterior-middle cingulate cortex (aMCC), left dorsal lateral prefrontal cortex (dlPFC) and left inferior parietal gyrus (Figure 2, Table 2).

**Table 2:** Association between personal distress and strength of functional connectivities between dmPFC and other regions.

				MNI coordinate		
	Regions	*k*	*T*	*x*	*y*	*z*
Positive correlation	Left posterior insula	121	4.45	−42	−15	9
	Left occipital cortex	485	4.94	−18	−57	−24
	Right occipital cortex	260	4.94	33	−93	−6
	Occipital cortex	271	4.78	0	−66	−3
Negative correlation	Anterior middle cingulate cortex	66	4.04	3	6	27
	Left dorsal lateral prefrontal cortex	105	3.71	−21	39	42
	Left inferior parietal gyrus	113	4.64	−36	−81	36

## Discussion

The current study examined relationships between trait measure of personal distress and gray matter volume (using the VBM analysis) and resting-state FC (using the seed-based FC analysis) in the brain. The VBM analysis showed that personal distress was negatively associated with GM volume in dmPFC. The FC analysis showed that personal distress was positively associated with FC of the dmPFC with the left posterior insula and occipital cortex; and was negatively related to FC of the dmPFC with the aMCC, dlPFC and left inferior parietal gyrus.

GM volume in the dmPFC was found to be negatively correlated with personal distress in the current study. Researchers have supposed that dmPFC plays an important role in processing social information such as in the mentalizing process (Van Overwalle, 2009; Schurz et al., 2014). For example, dmPFC was activated when subjects made judgments about another person’s emotional states (Ochsner et al., 2004), and dmPFC activation was stronger in the task about others than in the task about self (Gallese et al., 2004; Jackson et al., 2006; Murray et al., 2012). These results suggest essential functions of dmPFC in distinguishing whether the subjective feeling is triggered by the others or oneself. Our results further suggested that decreased GM volume in dmPFC may reflect a declining ability in self-other distinction, which would then result in higher personal distress scores.

In the present resting-state FC analysis, personal distress was found to be positively correlated with connectivity between the dmPFC and left posterior insula. The insula is thought to be an important brain structure in sensory, affective and cognitive functions. GM volume in insula has been found to be positively correlated with personal distress scores in previous study (Banissy et al., 2012) and similar results were also observed using other questionnaires (Mutschler et al., 2013; Eres et al., 2015). Insula can be divided into an anterior part (AI) and a posterior part (posterior insula), and the former is supposed to process information about others’ affective feeling whereas the latter is thought to process primary interoceptive representation and is important for being a sentient self (Craig, 2009, 2010; Taylor et al., 2009). Given that the dmPFC is considered to process information about others while the posterior insula is supposed to process self-oriented information, in current study increased connectivity between the dmPFC and left posterior insula was associated with stronger personal distress, suggesting that connectivity between the dmPFC and left posterior insula might be in line with the suggestion that people treat other-oriented affective feelings as self-oriented.

The present resting-state FC analysis also showed that personal distress was negatively correlated with connectivity between the dmPFC and aMCC, dlPFC and left inferior parietal gyrus. The cingulate cortex is known as an important region in affective monitoring and cognitive control. Activation of aMCC was also robust during empathy for pain (Lamm et al., 2011) and for the other’s negative emotion such as disgust (Prehn-Kristensen et al., 2009). dlPFC was supposed to take part in emotion regulation processes (Fan et al., 2011; Etkin et al., 2015). Further, aMCC, dlPFC and inferior parietal gyrus are parts of the cognitive executive control network that is responsible for executive functions including initiation, inhibition, working memory, flexibility, planning and vigilance and response preparation (Cole and Schneider, 2007; Niendam et al., 2012). Thus, the connectivities between dmPFC and these regions might be involved in the regulation of negative affect, which is consistent with the current finding of the negative association between FC and personal distress.

Taken together, our results demonstrated that personal distress was negatively associated with GM volume in the dmPFC; was positively associated with FC between the dmPFC and posterior insula; and was negatively associated with functional connectivities between the dmPFC and aMCC, dlPFC and left inferior parietal gyrus. The findings suggest that personal distress is associated with how people process the source of negative affect during empathy. Future research is required to clarify the relationship between neural mechanisms of personal distress assessed by trait measure and specific tasks, and to address the neural substrates of personal distress in other groups, for example, clinical social workers.

## Data Availability

The data generated during and/or analyzed during the current study are available from the corresponding author on reasonable request.

## Author Contributions

SZhong, SL and XW designed the study and wrote the manuscript; SZhong, XX and HZ collected questionnaire data; SZhong, LG, JY and YH collected MRI data; SL, SZhong, YZ and CW analyzed MRI data; SZhong and SZheng analyzed questionnaire data. All authors commented on the manuscript.

## Conflict of Interest Statement

The authors declare that the research was conducted in the absence of any commercial or financial relationships that could be construed as a potential conflict of interest.

## Footnotes

^1^http://www.fil.ion.ucl.ac.uk/spm/software/spm12/

^2^http://dbm.neuro.uni-jena.de/cat12/

^3^http://www.fil.ion.ucl.ac.uk/spm/
